# Subacute and chronic, non-specific back and neck pain: cognitive-behavioural rehabilitation versus primary care. A randomized controlled trial

**DOI:** 10.1186/1471-2474-9-172

**Published:** 2008-12-30

**Authors:** Odd Lindell, Sven-Erik Johansson, Lars-Erik Strender

**Affiliations:** 1 Center for Family and Community Medicine, Karolinska Institutet, Alfred Nobels allé 12, SE-141 83 Huddinge, Sweden

## Abstract

**Background:**

In the industrial world, non-specific back and neck pain (BNP) is the largest diagnostic group underlying sick-listing. For patients with subacute and chronic (= full-time sick-listed for 43 – 84 and 85 – 730 days, respectively) BNP, cognitive-behavioural rehabilitation was compared with primary care. The specific aim was to answer the question: within an 18-month follow-up, will the outcomes differ in respect of sick-listing and number of health-care visits?

**Methods:**

After stratification by age (≤ 44/≥ 45 years) and subacute/chronic BNP, 125 Swedish primary-care patients were randomly allocated to cognitive-behavioural rehabilitation (rehabilitation group) or continued primary care (primary-care group). Outcome measures were *Return-to-work share *(percentage) and *Return-to-work chance *(hazard ratios) over 18 months, *Net days *(crude sick-listing days × degree), and the number of *Visits *(to physicians, physiotherapists etc.) over 18 months and the three component six-month periods. Descriptive statistics, Cox regression and mixed-linear models were used.

**Results:**

All patients: *Return-to-work share *and *Return-to-work chance *were equivalent between the groups. *Net days *and *Visits *were equivalent over 18 months but decreased significantly more rapidly for the rehabilitation group over the six-month periods (*p *< .05). Subacute patients: *Return-to-work share *was equivalent. *Return-to-work chance *was significantly greater for the rehabilitation group (hazard ratio 3.5 [95%CI1.001 – 12.2]). *Net days *were equivalent over 18 months but decreased significantly more rapidly for the rehabilitation group over the six-month periods and there were 31 days fewer in the third period. *Visits *showed similar though non-significant differences and there were half as many in the third period. Chronic patients: *Return-to-work share, Return-to-work chance *and *Net days *were equivalent. *Visits *were equivalent over 18 months but tended to decrease more rapidly for the rehabilitation group and there were half as many in the third period (non-significant).

**Conclusion:**

The results were equivalent over 18 months. However, there were indications that cognitive-behavioural rehabilitation in the longer run might be superior to primary care. For subacute BNP, it might be superior in terms of sick-listing and health-care visits; for chronic BNP, in terms of health-care visits only. More conclusive results concerning this possible long-term effect might require a longer follow-up.

**Trial registration:**

NCT00488735.

## Background

In Sweden, as all over the industrial world, back and neck pain is the largest diagnostic group underlying sick-listing [1]. The vast majority consists of non-specific back and neck pain (BNP) that requires no specific surgical, rheumatological or neurological treatment [2].

As 93% of the societal costs of back and neck pain are connected with sick-listing [3], return-to-work is crucial [4]. However, there is a lack of consistency and comprehensiveness in return-to-work measurements [5]. While earlier studies compared the return-to-work share at a specific time point, for example one year after baseline [6], later research has evaluated the time of return-to-work in survival analyses [7,8]. Another important issue is the health-care utilization needed to achieve certain treatment results. In that respect, a frequently-used outcome measure is the number of health-care visits [9,10].

Concerning treatment of BNP, the 1990s saw a breakthrough for the biopsychosocial model, which pinpoints time off work as an important disabling factor. Acute, subacute and chronic BNP are defined as BNP with full-time sick-listing for 0 – 21 days, 22 – 84 days and more than 12 weeks, respectively [11]. Acute BNP is managed by continuing ordinary activities as normally as possible, and manipulation if necessary. In cases of subacute and chronic BNP, multidisciplinary rehabilitation should be considered [12]. Multidisciplinary treatment includes a physician's consultation in addition to psychological, social or vocational intervention or a combination of these [13]. The three key components of successful multidisciplinary rehabilitation programmes for BNP are: reactivation and progressive increase in activity levels, addressing dysfunctional beliefs and behaviour by a cognitive-behavioural therapeutic approach, and occupational interventions [4]. Concerning back pain, programmes including these items have shown good results in several studies [7,14-17]. Randomized controlled trials have concerned patients with subacute back pain only [7-9,14,15,17,18], mixed groups with subacute or chronic back pain [16,19] or patients with chronic back pain only [20]. There is a serious lack of evidence concerning the rehabilitation of neck pain [13]. We have found no randomized controlled trial in which the same programme was offered to patients who were stratified by subacute and chronic BNP.

The high frequency of relapses after rehabilitation of BNP is associated with inadequate follow-ups. A short program might fail to achieve long-standing behavioural changes [21]. In the 1990s the vast majority of rehabilitation programs in Sweden were comparatively short, with a fixed duration averaging six weeks [22].

Primary care is the appropriate source of treatment for BNP [12]. In Sweden, however, notwithstanding clinical guidelines, only a small minority of individuals with subacute and chronic BNP receive multidisciplinary rehabilitation [23]. One reason might be the relative lack of family doctors. While the total number of Swedish physicians meets international standards, there are proportionately fewer physicians within primary care: the density of family doctors is .5 per 1000 population, compared with an OECD (Organisation for Economic Co-operation and Development) average of .8 [24].

Our project started in 2000 with the aim of comparing a multidisciplinary programme of cognitive-behavioural rehabilitation for subacute and chronic BNP with primary care. The specific aim of this study was to answer the question: within an 18-month follow-up, will the outcomes differ in terms of sick-listing and number of health-care visits?

## Methods

### Sick-listing in Sweden

In Sweden, publicly provided, tax-financed social insurance compensates loss of income due to illness. The ultimate decisions about sick-listing benefits, including sickness benefit, rehabilitation benefit, temporary disability pension and disability pension, are made by the Social Insurance Agency. For sick-listing exceeding seven calendar days, a physician's certificate is required. The certificate comprises a detailed description of symptoms and signs and a recommendation of the degree (.25, .50, .75 or 1.00 (= full-time)) and duration of sick-listing.

### Participants

The rehabilitation centre of this study was situated at Haninge, a municipality 25 kilometres south-east of Stockholm city. As the centre was well known to the local residents, the study participants were recruited within the primary care of the adjoining municipalities. One-hundred-and-twenty five patients were recruited by 42 family doctors at 12 health centres.

*The criteria for inclusion: *1. Working age up to and including 59 years. 2. Sick-listed full-time for BNP at least six weeks (42 days) and at most two years (730 days). 3. Able to fill in forms. *The criteria for exclusion: *1. Temporary disability pension or disability pension being paid or in preparation. 2. A primary need for a hospital specialist (for example, operation for slipped disc). 3. Pregnancy and diseases (other than BNP) that might make rehabilitation impracticable (for example, advanced pulmonary disease). 4. Whiplash-associated disorders as a primary obstacle to working. 5. Previous rehabilitation at the rehabilitation centre. 6. Other multidisciplinary rehabilitation current or planned.

### Interventions

One treatment group was allocated to cognitive-behavioural rehabilitation at the rehabilitation centre (rehabilitation group). The other treatment group was allocated to continued primary care (primary-care group).

#### Cognitive-behavioural rehabilitation

The rehabilitation centre was opened in 1991 within Stockholm County Council. From 2002 it operated as a private company and the number of rehabilitation teams was decreased from four to one, comprising four team members: a physician (OL), a physiotherapist trained in manual therapy, a psychologist or a social worker trained in cognitive-behavioural therapy and a health-care adviser. Manual therapy includes manipulation, mobilisation and stabilizing training [25]. The centre used a cognitive-behavioural programme with the aim of achieving the maximal degree of work ability lasting for at least 30 consecutive days. Work ability was inversely proportional to sick-listing, which is the definition used by the Social Insurance Agency. Work abilities of 1.00 (= full-time), .75, .50 and .25 corresponded to sick-listings of 0, .25, .50 and .75, respectively. Zero work ability equalled full-time sick-listing. Possible relapses were met by individual and, when needed, long rehabilitation periods. The program is described in Table 1.

**Table 1:** Cognitive-behavioural rehabilitation.

**Staff category**	***Investigation and treatment phase, 2 – 8 weeks***	**Frequency**
Physician	Mapping out of medical obstacles to working. Handling of the sick-listing. If needed, prescription of drugs (antidepressants, analgesics etc.) and injections of cortisone (in shoulder- or hip-muscle attachments etc.)[25].	1 – 2 (consultations)/week.

Physiotherapist	Mapping out of biomechanical obstacles to working including a visit to the work place [14]. Start of graded activity: the patient first carried out an activity measurable in minutes, metres, etc., for example a walk, until the pain increased. The starting level was about 25% below that. A gradual increase of the activity was decided on check-ups, the final aim being to manage the load in a job, for the unemployed an imaginary one [14]. If needed, manual therapy [25].	2 – 3 consultations. 1/week. 1/week.

Psychologist or social worker	Mapping out of psychosocial obstacles to working. Cognitive- behavioural therapy focussed on anxiety and depression [46].	1/week.

Health-care adviser	Start of education in applied relaxation [46].	1/week for 6 – 8 w.

	***Action phase, 2 – 8 months***	

Team	Conference that produced a written rehabilitation plan with: 1.* Final aim *= the optimal degree of work ability that could be achieved and maintained for at least 30 consecutive days. *2. Partial aims *concerning functioning only (for example, increase of vocational training by five hours/week); symptom aims, for example, pain reduction, were excluded [14]. *3. Means of reaching the aims *(for example, increase of vocational training 1/2 hour/day week 1, 1 hour/day w. 2 etc.).	At the start of the action phase.

Team	Check-up conferences produced fresh partial aims.	1/3 – 4 weeks.

Team member (usually the physiotherapist)	Vocational conferences with the employer and a clerk from the Social Insurance Agency or, for unemployed patients, the Employment Office.	

Physician	Handling of the sick-listing.	1/3 – 4 weeks.

Physiotherapist	Completion of graded activity. Check-ups less frequent.	1/3 – 4 weeks.

Health-care adviser	Completion of education in applied relaxation.	1/week (f. 6 – 8 w.)

Psychologist or social worker	If needed: cognitive-behavioural therapy as support during the re-training process.	1/week.

	When the final aim was reached, or when it was obvious that return-to-work would not be achieved.	The end of rehabilitation.

Participation in the rehabilitation group did not exclude the patient from seeking other care, including primary care, during the follow-up period.

#### Primary care

The hubs of Swedish primary care are the health centres. They serve the local population and cater to its needs, with no restrictions as to illness, age or patient category, for basic medical treatment, nursing, preventive work or rehabilitation that does not require the medical and technical resources of hospitals or other special competences [26]. Most primary care in Sweden is publicly provided. Only a quarter is privately conducted [27]. Overall medical responsibility belongs to the family doctor. The 12 health centres in this study were situated in the municipalities of Tyresö, Huddinge, Stockholm and Nynäshamn. Ten of the centres were publicly provided, two were private. In total, they engaged 84 family doctors and served a population of 148,000 individuals, equivalent to barely .6 family doctors per 1000 population. Besides family doctors, their staff consisted of physiotherapists, nurses, assistant nurses, occupational therapists and social workers. Besides management at the health centre, primary care could include referral to consultation by, for example, an orthopedist or a neurologist.

Participation in the primary-care group excluded the patient from turning to the rehabilitation centre during the follow-up period but not from any other health-care, including multidisciplinary rehabilitation at units other than the rehabilitation centre.

### Outcome measures

#### *Return-to-work share*

The percentage of patients who regained any degree of work ability for at least 30 days in succession over 18 months. This was the primary outcome measure. Secondary outcome measures were:

#### *Return-to-work chance*

The chance, as expressed in hazard ratios, of achieving any degree of work ability over 18 months, irrespective of the duration of that work ability.

#### *Net days*

Sick-listing, expressed in whole days, over 18 months and the three component six-month periods. *Net days *= crude days × degree [28].

#### *Visits*

The total number of health-care visits over 18 months and over the three component six-month periods. *Visits *comprised consultations at the rehabilitation centre, within primary care and other care, including alternative-care providers, but excluded consultations relating to multidisciplinary rehabilitation at units other than the rehabilitation centre.

### Analyses and statistics

Except for descriptive statistics [29,30], Cox regression and mixed-linear models were used.

*Return-to-work chance *was compared by a Cox regression analysis for recurrent events with event dependence and a time interaction with the exposure variable (i.e. rehabilitation group or primary-care group) and is presented as hazard ratios with 95% confidence intervals [31]. It was analysed at six, 12 and 18 months.

*Net days *and *Visits *in the first, second and third six-month periods were outcome variables in two separate mixed-linear models. In the models, the main effects of three explanatory variables and two interaction terms were compared using a random intercept model of the unstructured covariance type on the group level and time as repeated factor [32]. The explanatory variables were time (i.e. six-month period 1, 2 or 3), rehabilitation group or primary-care group, and subacute or chronic patient. The interaction terms were time × rehabilitation group or primary-care group and time × rehabilitation group or primary-care group × sub-acute or chronic. The models were also adjusted for possible baseline characteristics with significant differences between the groups. The analyses were performed using PROC MIXED in SAS, version 9.1, and the results are presented as separate graphs for the subacute and chronic patients and as means with 95% confidence intervals and *p*-values, adjusted for all parameters (main effect and interactions).

**Figure 1:**
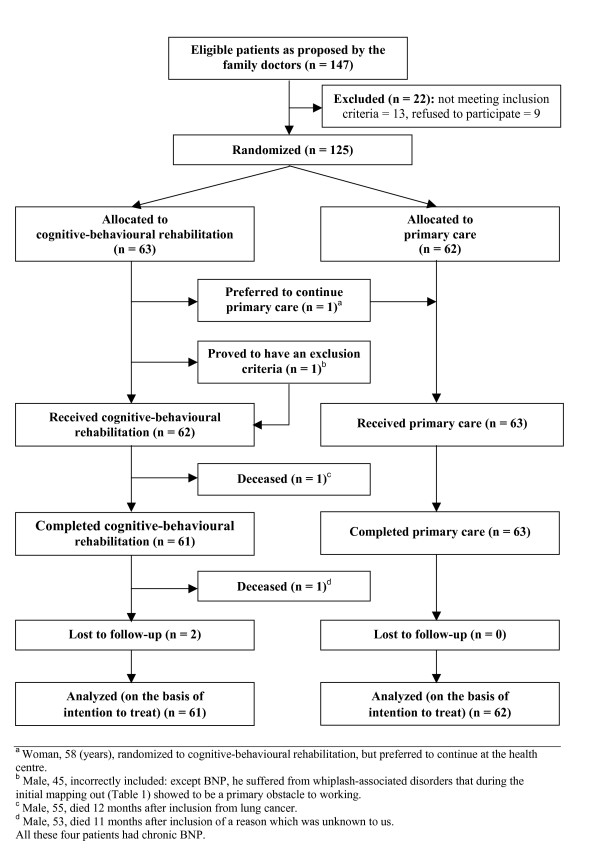
**Flowchart**.

The two patients who died (Figure 1) were excluded from the outcome analyses except from the Cox regression [31]. *Visits *at 18 months were analysed for those patients who had completed all the follow-up forms, while the mixed-linear model also included incomplete responders. To evaluate their possible influence on the treatment results, we also analysed the days of hospital care, the use of surgery for musculoskeletal disorders and multidisciplinary rehabilitation at units other than the rehabilitation centre.

The analyses were performed on an intention-to-treat basis. The primary outcome measure was also subjected to a per-protocol analysis [33]. The total percentage of withdrawals and drop-outs was calculated. This sum should not exceed 30% [34]. Baseline characteristics of responders and non-responders were compared. A *p*-value < .05 or, concerning the Cox regression, a 95% confidence interval not including 1.00, was considered statistically significant. Except for the mixed linear models, analyses were performed using Stata, 9.1.

### Blinding

The analyst of the sick-listing data was blind to the intervention alternative. Blinding was not possible for the other outcomes. For example, which of the two interventions was offered could not be concealed from either the care providers or the patients.

### Data collection

The sick-listing data were provided by the Stockholm County Social Insurance Agency. Data concerning the rehabilitation centre were collected from the medical records of the centre. Primary care and other health-care data were obtained from follow-up forms. Although these self-report measures have been used successfully in previous research, their reliability and validity have not been established. However, because the patients were free to seek treatment at any other facility, the only comprehensive sources of health-care data were self-ratings [9]. The data were fed into a specially designed database using Access version 2000.

### Power calculation

To calculate the power, a preliminary study was performed. In this retrospective study, 172 consecutive patients with subacute and chronic BNP, who completed rehabilitation at the centre during the period 1996 – 2000, were included. The mean rehabilitation period was 266 (SD ± 170) days. The *Return-to-work share *was 76%; for subacute and chronic BNP 89% and 73%, respectively (*p *< .05). The power calculation was based on this preliminary study and a forecast of the probability of return-to-work after traditional care for BNP [35]. The forecast probability for the patients in the preliminary study was calculated from their current sick-listing at baseline. It proved to be 49%, i.e. 27 percentage units less than the actual rate of 76%. Including an uncertainty about the application of this forecast to our patient sample, we expected to reach a difference between the rehabilitation group and the primary-care group of at least 22 percentage units. With an alpha of .05 and a power of 80%, this should require the inclusion of 154 patients; or, to allow a reasonable dropout rate, 170 patients.

### Inclusion procedure

For the patients who fulfilled the criteria, the family doctor gave verbal and written information about the project. Each patient who gave his or her oral consent to participate to the family doctor was interviewed by telephone by a research assistant within two days. The patients who still qualified for the study saw the assistant at the health centre within five days. At the appointment, the patient signed an informed consent to participate and went through an initial form including, among other items, the baseline characteristics in Table 2. Then the assistant carried out, among other tests, a lift test [36]. The reliability of that test procedure was confirmed in a separate study [37]. After stratification by age (≤ 44/≥ 45 years) and subacute/chronic BNP, the assistant performed the randomization. The two treatment alternatives were distributed in opaque envelopes by a computerized block-randomization procedure produced by an independent statistician. The assistant opened the remaining envelope with the lowest random number and presented the content to the patient.

**Table 2:** Baseline characteristics.

	Rehabilitation group (n = 63)	Primary-care group (n = 62)	*p*-value
Women	33 (52 [40 – 65]%)	35 (56 [44 – 69]%)	NS

Age (years)	42.2 [39.8 – 44.6]	43.0 [40.4 – 45.7]	NS

Neck-pain domination	17 (27 [16 – 38]%)	21 (34 [22 – 46]%)	NS

Widespread (= back + neck) pain	55 (87 [79 – 96]%)	45 (73 [61 – 84]%)	**.04**

Pain score (VAS, 0 – 100; median (IQR))[48]:			

"Just now"	61 (30)	53 (30)	NS

"Worst last week"	77 (29)	73 (26)	NS

Health-related quality of life (EQ-5D)[49]			

(median (IQR))	.489 (.332)	.497 (.332)	NS

Immigrants (= born outside Sweden)	19 (30 [19 – 42]%)	15 (24 [13 – 35]%)	NS

Single life	19 (30 [19 – 42]%)	21 (34 [22 – 46]%)	NS

Low education (= at most junior high school)	37 (60 [47 – 72]%)	35 (56 [44 – 69]%)	NS

Blue-collar work (of the non-unemployed)	41 (87 [77 – 97]%)	47 (87 [77 – 97]%)	NS

Unemployed	14 (22 [12 – 33]%)	15 (24 [13 – 35]%)	NS

Previous sick-listing (days)*	223 [189 – 257]	222 [188 – 256]	NS

Lifting capacity (kg; mean):			

PILE lumbar [36]	12.3 [10.4 – 14.2]	12.4 [10.3 – 14.6]	NS

PILE cervical [36]	11.5 [9.7 – 13.3]	11.6 [9.6 – 13.6]	NS

Descriptive statistics. The 95% confidence intervals are shown within brackets. Bold figures indicate a significant difference.

NS = Non-significant; IQR = Inter-quartile-range.

* = *Net days *over the 18 months preceding baseline.

### Ethical approval

Approval for the study was given by The Research Ethics Committee, Karolinska University Hospital, Huddinge.

### Premature cessation of recruitment

The recruitment of participants started in August 2000 and was discontinued in January 2004, when 125 patients were included. The reason was the opening in April 2004 of a large back-rehabilitation centre in a neighbouring municipality (Nacka) on the initiative of the Stockholm County Social Insurance Agency and Stockholm County Council. We presumed that many future study patients who would be randomized to the primary-care group would be referred to that centre and would contaminate the primary-care branch of our study.

### Follow-up

Six, 12 and 18 months after inclusion, the patients completed forms concerning, among other items, health-care utilization. If necessary, a postal reminder was sent after two weeks and a telephone reminder after another two weeks. If the forms were not returned despite these measures, the data were considered missing. The patient who was last to be included completed the 18-month follow-up period in July 2005.

## Results

### Response rate and missing data

Data for the baseline characteristics, sick-listing and care at the rehabilitation centre were complete. For other health-care data, the response rates for the six-, 12- and 18-month forms in the rehabilitation group (n = 61) were 57 (93%), 56 (92%) and 55 (90%) respectively and all forms were answered by 51 patients (84%). The corresponding rates for the primary-care group (n = 62) were 50 (81%), 48 (77%), 50 (81%) and 42 (68%). Non-responders and responders are compared in Table 3.

**Table 3:** Missing data.

Follow-up	Six months	*p*-value	12 months	*p*-value	18 months*	All forms	*p*-value
Rehabilitation group (n = 61)							
Previous sick-listing (days)**	397 vs. 215	.008	371 vs. 214	.01	-	-	
Current sick-listing at baseline (days)	367 vs. 158	< .001	346 vs. 156	< .001	-	275 vs. 151	.003
Unemployment (%)	-	-	60 vs. 18	.03	-	-	

Primary-care group (n = 62)							
Age (years)	35.8 vs. 44.8	.006	-		-	38.3 vs. 45.3	.01
Single (%)	58 vs. 28	.046	-		-	-	
EQ-5D [49]	-	-	.357 vs. .562	.046	-	-	

Non-responders versus responders. Significant differences at baseline. Descriptive statistics.

*At 18 months there were no significant differences.

** = *Net days *over the 18 months preceding baseline.

### Baseline characteristics and participant flow

Except for a higher prevalence of widespread pain in the rehabilitation group, there were no significant differences (Table 2). When analyzed separately (data not shown), the subacute rehabilitation-group patients were equal to the subacute primary-care-group patients while the chronic rehabilitation-group patients had a much higher prevalence of widespread pain: 93 [85 – 100]% versus 68 [54 – 82]% for the chronic primary-care-group patients (*p *= .004).

Patients who were allocated to the rehabilitation group started the programme within one week. Patients who were allocated to the primary-care group continued care at their health-centres. Sixty-one patients in the rehabilitation group completed cognitive-behavioural rehabilitation; all primary-care-group patients completed primary care (Figure 1). The two deceased rehabilitation-group patients had passed the "red-flags" examinations [12] at the start without remark.

### Outcome measures

#### *Return-to-work share*

There were no significant differences between the rehabilitation group and the primary-care group, or between the subacute and chronic patients considered separately (Table 4). In both the rehabilitation group and the primary-care group, most of the patients who regained any degree of work ability returned to full-time work: 20/35 (57%) and 25/35 (71%) respectively (non-significant). The mean degrees of work ability at return to work were .77 [.67 – .87] and .85 [.76 – .94] respectively (non-significant).

**Table 4:** *Return-to-work share, Net days *and *Visits*.

	Patients	Rehabilitation group	Primary-care group
*Return-to-work share *(%)	All	35/61 (57 [45 – 70])	35/62 (57 [44 – 69])
	Subacute	18/20 (90 [76 – 104])	15/18 (83 [64 – 102])
	Chronic	17/41 (42 [26 – 57])	20/44 (46 [30 – 61])

*Net days*	All	397 [354 – 440]	391 [345 – 436]
	Subacute	327 [261 – 392]	292 [194 – 391]
	Chronic	431 [377 – 486]	431 [383 – 478]

*Visits*	All	55.7 [49.3 – 62.2]	52.0 [38.1 – 66.0]
	Subacute	48.3 [38.5 – 58.1]	40.6 [23.1 – 58.1]
	Chronic	60.1 [51.6 – 68.7]	56.6 [38.1 – 75.2]

Point estimates at 18 months. Descriptive statistics.

#### *Return-to-work chance*

The hazard ratio for the rehabilitation group increased over the three six-month periods in comparison to the primary-care group, but the difference did not reach significance (Table 5). The subacute rehabilitation-group patients showed a substantial increase over these periods and achieved a significantly higher hazard ratio at 18 months than the subacute primary-care-group patients. There were no differences for the chronic patients.

**Table 5:** *Return-to-work chance*.

Rehabilitation group	Six months	12 months	18 months
All patients (n = 61)	.9 [.6 – 1.4]	1.2 [.7 – 2.0]	1.6 [.7 – 3.6]
Subacute patients (n = 20)	.9 [.5 – 1.6]	1.8 [.8 – 3.9]	**3.5 [1.001 – 12.2]**
Chronic patients (n = 41)	.9 [.5 – 1.6]	.9 [.4 – 2.1)	1.0 [.3 – 3.9]

Cox regression for recurrent events. Hazard ratios for the rehabilitation group as compared with the primary-care group with 95% confidence intervals. Significant differences in bold figures.

#### *Net days*

**Figure 2:**
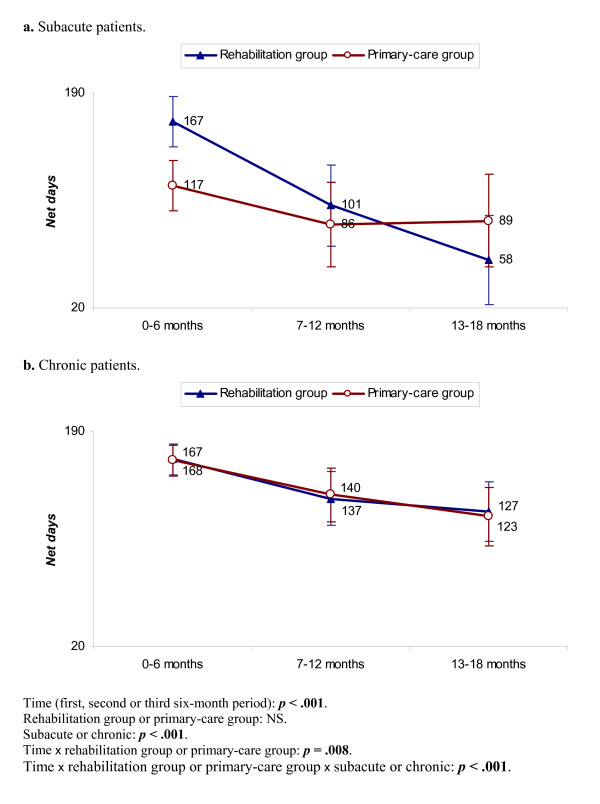
**a – b. *Net days***. Mixed linear model. In the diagrams, 95% confidence intervals are included. At the bottom the explanatory variables and their *p*-values are shown. Bold figures indicate a significant difference. NS = non-significant.

At 18 months there were no significant differences between the treatment groups, or between the subacute and chronic patients considered separately (Table 4). Over the three six-month periods, the decrease was significantly more rapid for the whole rehabilitation group and for the subacute rehabilitation-group patients considered separately (bottom of Figure 2a–b). In the first six-month period, there were 50 more *Net days *for the subacute rehabilitation-group patients; in the third period there were 31 days fewer (Figure 2a). There were no differences for the chronic patients (Figure 2b). Adjustment for widespread pain showed no changes.

#### *Visits*

**Figure 3:**
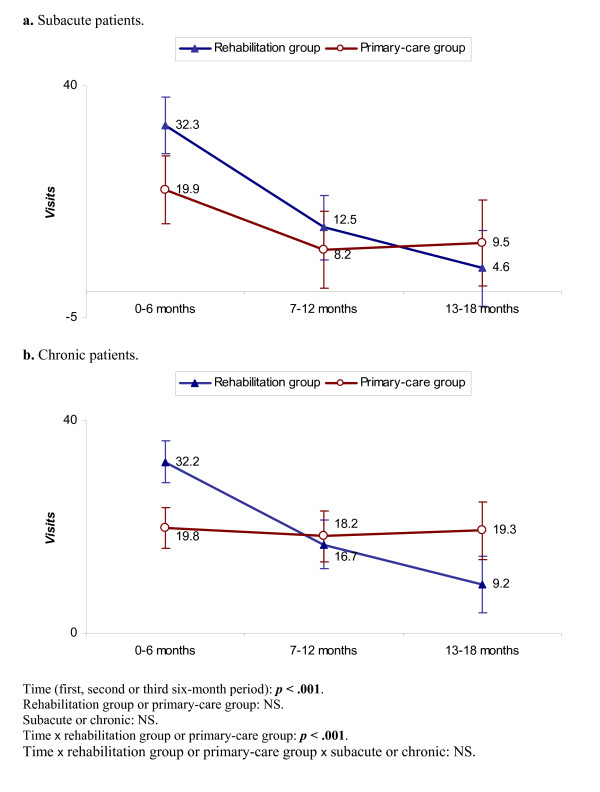
**a – b. *Visits***. Mixed linear model. Further explanations in Figure 2a–b.

At 18 months there were no significant differences between the treatment groups or between the subacute and chronic patients considered separately (Table 4). Over the three six-month periods, the decrease was significantly more rapid for the whole rehabilitation group (bottom of Figure 3a–b). For the subacute patients, the rehabilitation group showed a continuously decreasing trend while the primary-care group showed a substantial decrease between the first and second six-month periods but no further reduction (Figure 3a). For the chronic patients, the rehabilitation group showed a continuous decrease while the primary-care group showed no reduction (Figure 3b). *Visits *were substantially more numerous for both the subacute and chronic rehabilitation-group patients during the first period, but there were around half as many in the third period. However, there was no significant difference in the rate of decrease between the subacute and chronic patients considered separately (bottom of Figure 3a–b). Adjustment for widespread pain gave no changes.

### Interventions

#### Cognitive-behavioural rehabilitation

**Figure 4:**
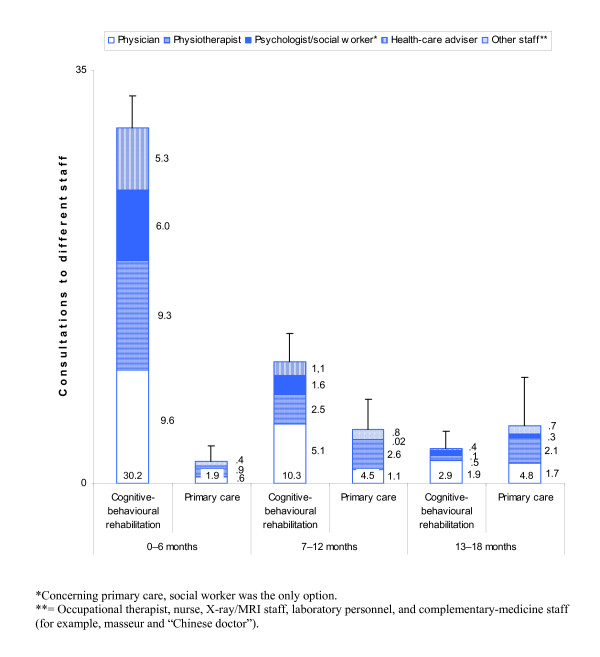
**Consultations to different care staff for the rehabilitation group**. For the total number (presented at the bottom of the staples), 95% confidence intervals (upper part) are shown.

Cognitive-behavioural rehabilitation over 18 months included 45.1 [39.2 – 50.9] consultations. Most of the consultations took place in the first six-month period, followed by a rapid reduction (Figure 4). Totalling 0 – 18 months, the most and second most frequent consultations were with a physician (16.6 [14.4 – 18.7]) and a physiotherapist (12.3 [10.5 – 14.1]). A detailed description of the rehabilitation programme is shown in Table 6.

**Table 6:** Cognitive-behavioural rehabilitation.

Rehabilitation period (days)	Total period 328 (± 195); median 283 (IQR215)	Investigation and treatment phase 42 (± 18); median 40 (IQR22)		Action phase 287 (± 193); median 249 (IQR232)
	Consultations			
	One-to-one	Treatment measure	At conferences	In total

Physician	7.3 (± 5.2)	Administration of sick-listing 61/61 (100%)	10.6 (± 6.8)	17.9 (± 11.0)
		Prescription of drugs 53/61 (87%)		
		Cortisone injections 9/61 (15%)		
				
Physiotherapist	7.8 (± 4.9)	Graded activity 61/61 (100%)	4.6 (± 3.4)	12.4 (± 7.1)
		Orthopaedic manual therapy 15/61 (25%)		
				
Psychologist or social worker	4.8 (± 5.2)	Cognitive-behavioural therapy 58/61 (95%)	3.4 (± 3.0)	8.2 (± 7.8)
Health-care adviser	6.2 (± 4.8)	Applied relaxation 48/61 (79%)	.3 (± .8)	6.6 (± 5.3)
				
Conferences:				
Team conferences	8.6 (± 5.7)			
Vocational conf. (incl. workpl. visits)	2.4 (± 2.4)	Vocational training 32/61 (52%)		
				
	__________			__________
Sum of treatment occasions	37.1 (± 19.2)		Sum of consultations	45.1 (± 22.8)

Physical activity (days/week):				
Exercise programme	5.5 (± 2.2)			
Gym training	1.0 (± 1.3)			

Specification of measures. Number of consultations (mean (SD)) unless otherwise stated.

SD = Standard deviation; IQR = Inter-quartile range.

#### Primary care

For the rehabilitation group, primary care over 18 months comprised 11.7 [6.7 – 16.7] consultations. After a slight increase from the first to the second six-month period, there was stagnation (Figure 4). During the first six-month period most of the rehabilitation-group patients (41/57 (72%)) had no primary-care consultations at all.

**Figure 5:**
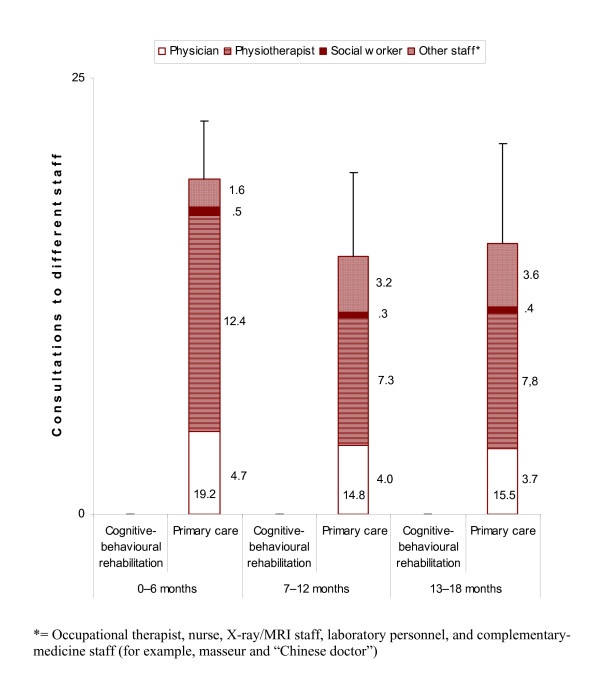
**Consultations to different care staff for the primary-care group**. Further explanations in Figure 4.

For the primary-care group, primary care over 18 months included 50.9 [37.5 – 64.3] consultations. After a slight decrease from the first to the second six-month period there was no further reduction (Figure 5). Totalling 0 – 18 months, the most and second most frequent consultations were with a physiotherapist (28.9 [19.4 – 38.4]) and a physician (12.4 [10.2 – 14.7]).

#### Other treatment efforts

Hospital care was received by the rehabilitation group and the primary-care group for 1.2 [-.2 – 2.6] days and .8 [.1 – 1.6] days respectively, surgery for musculoskeletal disorders by 1/51 (2 [-2 – 6]%) and 3/43 (7 [-1 – 15]%) respectively, and multidisciplinary rehabilitation at other units than the rehabilitation centre by 1/50 (2 [-2 – 6]%) and 4/43 (9 [0 – 18]%) respectively. The differences were non-significant.

### Per-protocol analysis

When the incorrectly included rehabilitation-group patient (Figure 1, footnote b) was excluded from the analyses and the rehabilitation-group patient who preferred to continue primary care (Figure 1) was counted with the primary-care group, the *Return-to-work share *increased to 44 [28 – 59]% for the chronic rehabilitation-group patients, and decreased to 44 [30 – 59]% for the chronic primary-care-group patients. This differed only marginally from the intention-to-treat analyses.

## Discussion

This randomized controlled trial concerned primary-care patients with subacute and chronic BNP. A programme of cognitive-behavioural rehabilitation was compared with continued primary care. The results were equivalent over 18 months. However, analyses of the three component six-month periods indicated that the rehabilitation programme might be superior to primary care in the longer run, especially for subacute patients.

### Sick-listing

Why was the *Return-to-work share *substantially lower than expected for the rehabilitation group and higher than expected for the primary-care group? According to Englund et al. [38], sick-listing in Swedish primary care might depend more on the patient's wishes than on guidelines: even when the family doctor did not recommend sick-listing, a certificate was issued in 87% of cases. In view of this, what explains the substantial underestimation of the *Return-to-work share *for the primary-care group (49% vs. the actual share of 57%)? One explanation might be a project that was initiated by the Swedish government in 2002 to halve the extent of sick-listing by 2008 [39]. The focus has been on applying more restrictions in the social insurance system, including failing an increasing number of sick-listing certificates, while the resources for multidisciplinary rehabilitation have been even scarcer than before [40,41]. Anyhow, the low *Return-to-work share *in the rehabilitation group was disappointing, and even if the primary-care group had shown as low a *Return-to-work share *as predicted, the difference between the groups would have remained non-significant.

However, when subacute and chronic patients were analysed separately, a different picture emerged: the *Return-to-work share *for the subacute rehabilitation-group patients was as expected, but the share for the chronic rehabilitation-group patients was far lower. The significantly better *Return-to-work chance *at 18 months and the more rapid reduction in *Net days *among the subacute rehabilitation-group patients highlighted this. Previous research supports the view that cognitive-behavioural interventions at an early stage of disabling BNP can prevent long-term disability [9,10,14,42], while the effect on sick-listing is more doubtful for chronic back pain. Schonstein et al. [43] concluded that physical conditioning programs with a cognitive-behavioural and work-related approach reduced sick-listing, whereas another Cochrane review revealed that behavioural-rehabilitation programmes had no better effect on sick-listing for chronic back pain than active conservative treatment [20].

What components of our programme could explain its possible superiority in the long run for subacute patients? Previous research on graded activity had an occupational-care setting and concerned subacute patients only [7,8,14,18,44,45]. Two earlier studies [7,14] found that graded activity in multidisciplinary contexts decreases sick-listing. Two later studies [18,44] contradicted that. Steenstra et al. [18] found that workplace interventions alone reduced sick-listing, while graded activity alone or in combination with workplace interventions did not. One explanation might be that the earlier studies were performed in specialised in-company clinics by a limited number of physiotherapists, including some of the researchers, while the study by Steenstra et al. also included out-company clinics with many physiotherapists who had received additional training [18]. These six-month results were confirmed at a 12-month follow-up [8]. Heymans et al. [44] found that standard care plus a low-intensity back school of eight hours was superior to standard care alone, while standard care plus a high-intensity graded-activity-like back school tended to be inferior. The follow-up period of those later studies did not exceed 12 months. In our study, however, the better sick-listing trend for the subacute rehabilitation-group patients was not obvious until after 12 months. Thus, the possibility that a longer period of graded activity has a positive effect on sick-listing for subacute patients in a primary-care setting could not be excluded from those later studies. As to the rest of our specific cognitive-behavioural elements (therapy by a psychologist or a social worker and training in applied relaxation), earlier conclusive studies are lacking [46].

Unlike previous research on graded activity, we also included chronic BNP. Most of the rehabilitation-group patients (43/63 (68%)) had a current sick-listing exceeding 12 weeks at baseline. Our programme did not reduce their sick-listing. Why? One reason could be its comparatively limited extent. Haldorsen et al. [16] showed that, for return-to-work, light multidisciplinary treatment was adequate for moderately-disabled but not for highly-disabled patients. For the latter group, extensive multidisciplinary treatment totalling 120 hours was required; the light programme was no better than standard care. Jensen et al. [19] showed that an extensive behavioural-rehabilitation programme (fully 120 hours) for long-term BNP in female patients reduced sick-listing while more limited efforts did not. Males, however, achieved no better results from the full-time programme than from a light programme or standard care. Quite recently, Staal et al. [45] found that moderately disabled subjects benefited more from graded activity than those with higher disability scores. These studies indicate that return-to-work for patients with chronic BNP, if it is ever possible, requires a more extensive concept than our programme.

Another reason could be methodological defects. Graded activity *by the book *includes: two sessions/week over a maximum of 3–6 months until lasting full-time return-to-work, an early agreement with the patient on a return-to-work date regardless of the actual pain on that particular day, and a hands-off approach [7,18]. As our patients were comparatively more disabled, we found it realistic to apply less frequent sessions to increase the likelihood of positive changes at the next session (there was also a lack of resources for more frequent sessions), no upper time limit (which is also in accordance with the original concept [14]), the possibility of part-time return-to-work, an individual agreement about the return date (early in the rehabilitation period for some patients, later for others) and, when needed, manual therapy and cortisone injections early in the rehabilitation period (however, the hands-off approach was applied to most (46/61 (75%)) of our patients). Notwithstanding the logical reasons for most of our modifications, they might have contributed to the failure to decrease the sick-listing of the chronic patients. These discrepancies might also explain why the positive effect on the subacute rehabilitation-group patients was not seen until the third six-month period, while those patients had substantially more *Net days *during the first period. It has recently been pointed out that suboptimal rehabilitation items in the pre-phase of return-to-work entail the risk of a counterproductive effect [18].

### Health-care visits

In total, the rehabilitation group had more consultations by a physician, which is more costly than other staff categories. However, the resources spent on the rehabilitation group in the first six-month period were balanced by fewer consultations in primary care and a trend towards fewer *Visits *in the long run. Also, although the differences were not significant, the rehabilitation group tended to experience less surgery and other multidisciplinary rehabilitation. For patients with subacute BNP, this agrees with Linton et al. [9], whose cognitive-behavioural interventions were followed by a decrease in health-care utilization. For patients with chronic BNP, our findings are consistent with a large review showing that cognitive-behavioural programs have a substantial positive impact on psychological and medical function but only a small impact on sick-listing [46].

### Strengths of the study

The design of our study, a randomized controlled trial, is the gold standard for evaluating treatment methods for back and neck pain [2].

The sick-listing data were complete. We also consider the health-care data to be acceptably representative. The response rate was higher than 80% except at 12 months, when it was nearly 80% for the primary-care group. Even when the missing data for the two deceased patients were included, the rehabilitation group met drop-out criteria [34]. For the primary-care group, *Visits *over 18 months should be interpreted with some caution as 32% were non-responders, but in other respects the follow-up rate of the primary-care group was also satisfactory. The non-responders in the rehabilitation group had characteristics that may have increased health-care use (longer sick-listing periods and higher unemployment). In the primary-care group the non-responders were younger, which could have decreased utilization, whereas the lower health-related quality of life could possibly increase utilization. However, for the great majority, there were no significant differences at baseline between the non-responders and responders.

### Limitations of the study

The inclusion plan was not fulfilled. A possible consequence may have been that some differences between the groups could not be demonstrated. However, certain differences in favour of the rehabilitation group were clear with the number of patients actually included.

Comparison of health-care visits gives only a limited idea of cost effectiveness. A complete health-economic evaluation is planned in a future study, including a cost-benefit analysis in which the direct costs (mainly of the interventions themselves), the indirect costs (mainly of the sick-listing), and the health-related quality of life are compared [47].

The primary outcome measure showed no difference. Notwithstanding the positive trends in favour of the rehabilitation group, especially for the subacute patients, *Net days *and *Visits *were also equivalent over 18 months. As differences in the results of various interventions tend to even out after 12 – 18 months [19], more conclusive results might require a longer follow-up period than in this study.

## Conclusion

For patients with subacute and chronic BNP, cognitive-behavioural rehabilitation was compared with primary care. The results were equivalent over 18 months. However, there were indications that cognitive-behavioural rehabilitation in the longer run might be superior. For subacute BNP, it might be superior in terms of both sick-listing and health-care visits; for chronic BNP, in terms of health-care visits only. More conclusive results concerning this possible long-term effect might require a longer follow-up.

## Competing interests

The authors declare that they have no competing interests.

## Authors' contributions

OL was the main investigator, carried out the study, performed the analysis, and drafted the manuscript. SEJ contributed to the statistical analysis. LES, as supervisor for OL, participated in all phases of the study. All authors read and approved the final manuscript.

## Pre-publication history

The pre-publication history for this paper can be accessed here:


